# Systemic blockade of P2X7 receptor protects against sepsis-induced intestinal barrier disruption

**DOI:** 10.1038/s41598-017-04231-5

**Published:** 2017-06-29

**Authors:** Xiuwen Wu, Jianan Ren, Guopu Chen, Lei Wu, Xian Song, Guanwei Li, Youming Deng, Gefei Wang, Guosheng Gu, Jieshou Li

**Affiliations:** Department of Surgery, Jinling Hospital, Medical School of Nanjing University, Nanjing, China

## Abstract

Sepsis, during which the intestinal epithelial barrier is frequently disrupted, remains a challenging and life-threatening problem in clinical practice. The P2X7 receptor (P2X7R) is a non-selective adenosine triphosphate-gated cation channel present in macrophages that is involved in inflammatory responses. However, little is known about the role of P2X7R in macrophages during sepsis-induced intestinal barrier disruption. In this study, mice were treated with the P2X7R antagonist A740003 or the agonist BzATP by intra-peritoneal injection after the induction of gut-origin sepsis. The survival rates, inflammatory responses, intestinal barrier integrity, macrophage marker expression, and ERK and NF-κB activities were evaluated. Intestinal macrophages were also isolated and studied after exposure to Brilliant Blue G or BzATP. We found that a systemic P2X7R blockade downregulated sepsis-induced inflammatory responses and attenuated intestinal barrier dysfunction based on the evidence that mice in the A740003-treated group exhibited alleviated pro-inflammatory cytokine synthesis, intestinal hyperpermeability, epithelial apoptosis rates and tight junction damage compared with the septic mice. These changes were partly mediated by the inhibition of M1 macrophages activation via ERK/NF-κB pathways. Our data presented herein show that a P2X7R blockade could be a potential therapeutic target for the treatment of sepsis-induced intestinal barrier dysfunction.

## Introduction

Sepsis remains a challenging and often life-threatening problem in clinical practice that is associated with unacceptable morbidity and mortality rates. With improved understanding of its pathobiology, sepsis is currently defined as life-threatening organ dysfunction caused by a dysregulated host response to infection^1^. The systemic and uncontrolled immune activation that is triggered by sepsis often leads to shock, multiple organ failure and death^2^. The intestinal barrier, which can prevent intestinal flora leakage beyond the gut, is frequently disrupted during sepsis. Breaking down or overwhelming this barrier may provide an outlet for viable bacteria and their antigens to move to other locations, leading to the development or aggravation of sepsis^3^. Hence, maintenance or repair of the intestinal barrier could be a target for sepsis prevention and treatment.

Some pro-inflammatory cytokines, such as IL-6 and TNF-α, have been found to contribute to the disruption of intestinal epithelial barrier function^4^. Macrophages are key effector cells of innate immunity and play important roles in sepsis resolution; however, they also contribute to aggravating the disease severity. In addition, macrophages can recognize microbes, initiate the inflammatory process, kill bacteria and secrete chemokines to attract other cells to inflamed sites^5, 6^.

The “classically activated” macrophages (M1 macrophages), which produce pro-inflammatory cytokines and effector molecules, are involved in the regulation of T-helper (Th) 1 cells and acute inflammation. In contrast, the “alternatively activated” macrophages (M2 macrophages) express large amounts of IL-10 and scavenger-, mannose- and galactose-type receptors, thereby activating Th2 cells and regulating extracellular matrix molecule synthesis^7^. Macrophages can undergo M1 or M2 activation upon exposure to a pathogen or a cytokine microenvironment^8^. Generally, macrophages are mainly exhibited as the M1 phenotype during the early stages of bacterial infection. If a macrophage-mediated inflammatory response cannot be quickly controlled, a cytokine storm occurs, thereby contributing to the pathogenesis of severe sepsis^9^. In addition, M2 macrophages activation could render anti-inflammatory effects and cause immunosuppression, which leads to secondary infection after sepsis and confers an even worse outcome^10^.

Recent evidence suggests that purinergic signaling is required for the development of the sepsis-associated inflammatory response. The P2X7 receptor (P2X7R) is an extracellular ATP-gated cation channel expressed in epithelial cells and immune effector cells and is involved in the regulation of pro-inflammatory cytokine production^11^, apoptosis and autophagy induction^12^, and host defenses against infectious pathogens^13^. In the absence of this receptor, septic mice exhibited improved survival, decreased levels of NO and pro-inflammatory cytokines, reduced peritoneal cell apoptosis, and produced less pronounced morphological changes^14, 15^. Despite the importance of P2X7R during inflammatory responses to pathogens, it is not established whether this receptor plays a role in sepsis-induced intestinal barrier dysfunction.

Here, we use a murine model to investigate the role of P2X7R on macrophages during sepsis-induced intestinal barrier dysfunction. We hypothesize that blocking this receptor will attenuate the sepsis-associated innate immune response and result in increased survival, reduced inflammatory burst, and enhanced intestinal barrier integrity. Additionally, the limitation of inflammatory responses following P2X7R repression may be associated with macrophage activation.

## Results

### Systemic blockade of P2X7R with A740003 blunts P2X7R expression and does not affect survival

**Figure 1:**
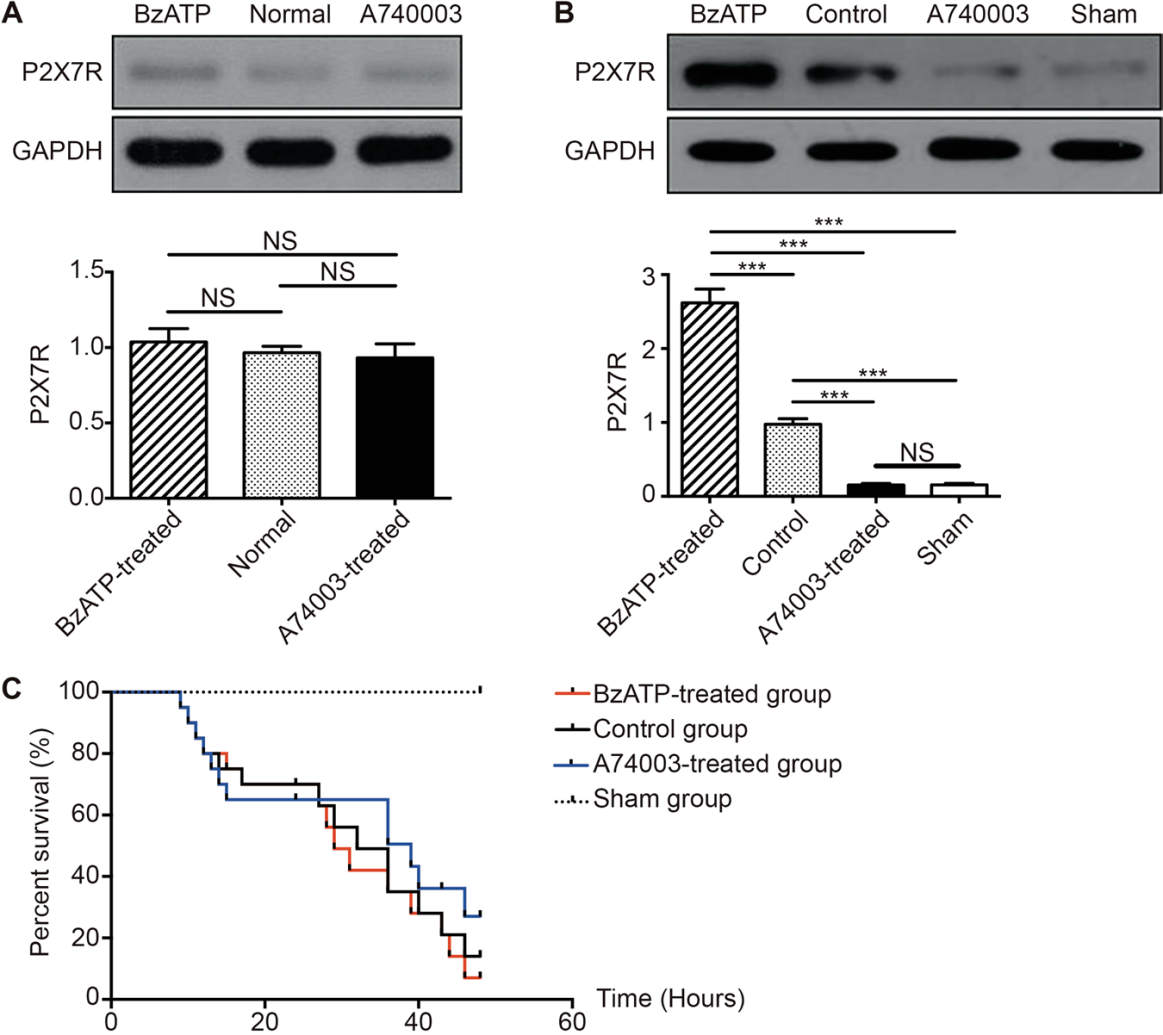
The effects of a P2X7R blockade on receptor expression and survival rates. The protein levels of P2X7R in intestines from mice that received intraperitoneal injections of BzATP or A740003 before CLP procedures (**A**) and 48 hours after CLP procedures (**B**) measured by WB. N = 6 mice per group. The results of the WB analysis are combined data from three densitometric scans (mean ± SD). (**C**) The Kaplan-Meier survival curve 48 hours after CLP. Twenty-four hours after CLP, mice received saline (control group, N = 20), BzATP (BzATP-treated group, N = 20), or A740003 (A740003-treated group, N = 20). The mice in the sham control group that underwent sham surgery (sham group, N = 6) were intravenously injected with saline. The Kaplan-Meier survival curves were compared by the log-rank test. *p < 0.05, **p < 0.01 and ***p < 0.001 when compared among the different groups.

First, we detected P2X7R expression in the intestines of mice that received an intraperitoneal injection of the agonist or antagonist before and after cecal ligation and puncture (CLP) procedures (Fig. 1A and B). Western blot (WB) analysis showed that there were no significant differences in P2X7R levels between the agonist/antagonist-treated mice and the normal mice before CLP intervention (p > 0.05). However, after CLP induction, a dose of 5 mg/kg BzATP significantly promoted P2X7R expression in the intestines compared with intestines in the sham group (p < 0.001) and the control group (p < 0.001). A740003, at a dose of 30 mg/kg, significantly lowered the expression of P2X7R compared with the control group (p < 0.001). There were no differences in the P2X7R levels between the A740003-treated group and the sham group (p > 0.05).

To evaluate the potential therapeutic efficacy of the P2X7R blockade, we used a severe model of polymicrobial sepsis (CLP) without antibiotics to produce high mortality during the early phase. With this procedure, the onset of clinical symptoms in C57BL/6 mice, such as piloerection and inactivity, were observed 6–12 hours after induction. As shown in Fig. 1C, a drop in survival rates ranging from 8–10 hours to 16 hours was found in all three CLP groups, and it was during this period that the maximum host immune responses, including bacterial dissemination and inflammation, occurred^16^. At 48 hours, mice in the BzATP-treated group, control group, and the A740003-treated group exhibited mortalities of 91%, 86%, and 73%, respectively. No significant differences were observed between the BzATP-treated/A740003-treated mice and the CLP mice (p > 0.05).

### Systemic blockade of P2X7R with A740003 suppresses pro-inflammatory cytokine production

Next, we investigated whether increasing or blocking P2X7R activity in septic mice could affect the production of inflammatory mediators involved in sepsis and intestinal inflammation (Supplementary Fig. S1). BzATP injection remarkably increased IL-6 and TNF-α expression in serum compared with levels in the sham group (p < 0.001) and control group (p < 0.001). In animals treated with A740003, serum levels of IL-6 were significantly lower than those in the sham group (p < 0.05) and the control group (p < 0.001). The levels of TNF-α were significantly reduced compared with those of control animals (p < 0.001), demonstrated by a 6–10-fold decrease, but there were no differences in the levels of TNF-α between the A740003-treated group and the sham group (p > 0.05). There was no difference in the serum production of anti-inflammatory cytokines, such as IL-10 and IL-13, between the A740003-treated/BzATP-treated mice and the control mice (p > 0.05). Compared with the sham group, the IL-10 levels were increased in the BzATP-treated group (p < 0.01) and decreased in the A740003-treated group (p < 0.001). BzATP did not evoke IL-13 expression, and A740003 treatment caused significantly reduced levels compared with the sham group (p < 0.001). Cytokine expression levels in intestinal tissue were similar to those in circulation.

### Systemic blockade of P2X7R with A740003 protects against intestinal barrier disruption

Because the above results showed that activation or inactivation of P2X7R could affect inflammatory responses, intestinal barrier function was evaluated among the groups to investigate its role in the development of polymicrobial sepsis.

#### Evaluation of intestinal injuries

**Figure 2:**
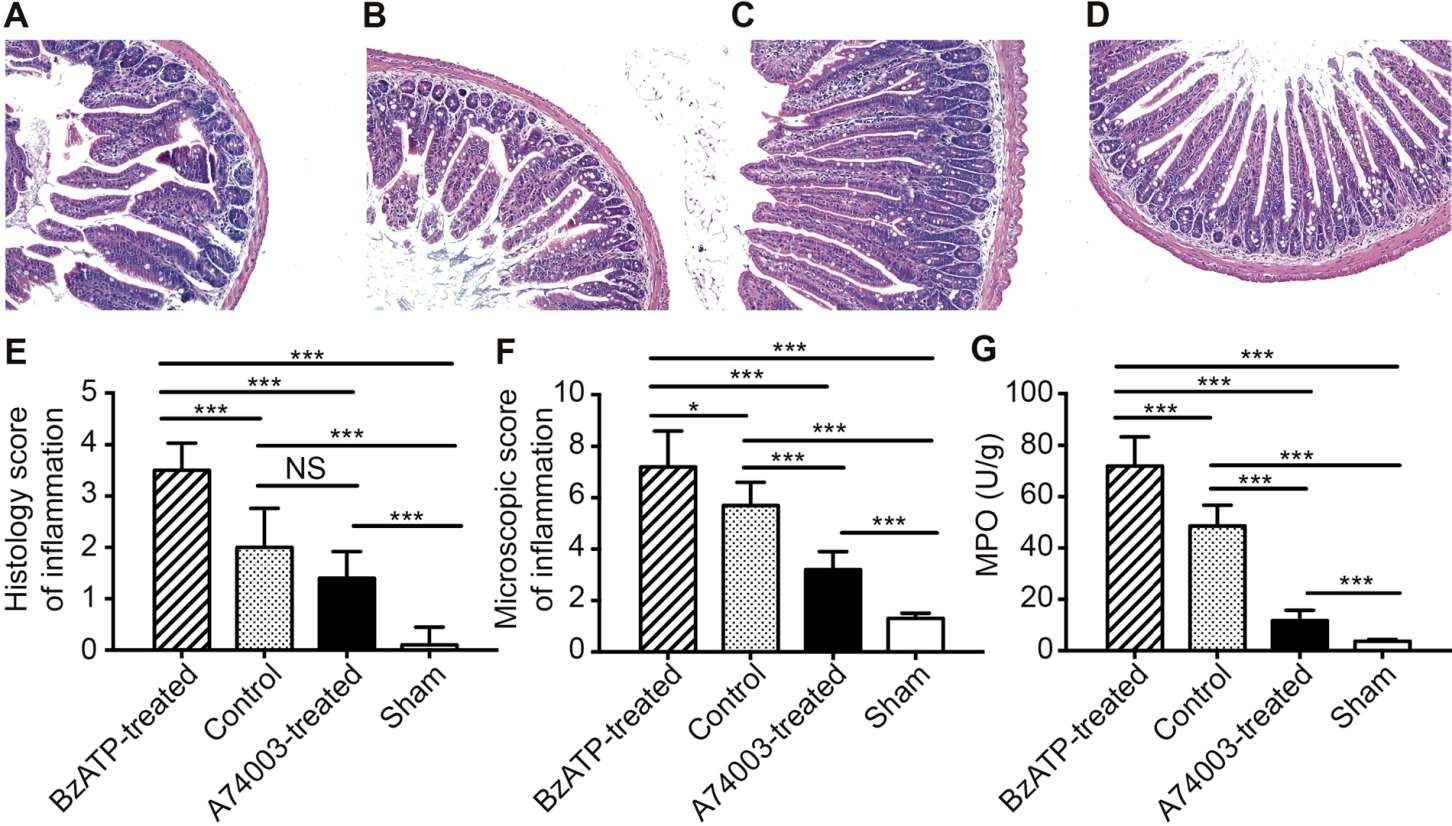
The effects of a P2X7R blockade on intestinal injuries. Intestinal tissues were obtained 48 hours after sepsis induction to evaluate the severity of inflammation. Representative H&E-stained intestinal sections in the BzATP-treated (**A**), control (**B**), A740003-treated (**C**), and sham (**D**) groups. The histological scores and macroscopic scores are shown in (**E**,**F**). (**G**) The intestinal samples of animals from each group were homogenized and protein was extracted for MPO activity analysis. N = 6 mice per group. The data are expressed as the means ± SD. *p < 0.05, **p < 0.01 and ***p < 0.001 when compared among the different groups.

Histopathologic evaluation of stained tissue sections from each group showed that the BzATP-treated group and control group exhibited impaired intestinal villi, severe inflammatory cell infiltration, and locally necrotic areas (Fig. 2A–D). A microscopic evaluation of intestinal injuries was conducted for each group (Fig. 2E). The BzATP-treated group had significantly increased mean histological scores compared with those of the sham group (p < 0.001) and control group (p < 0.001). The histologic evaluation of the A740003-treated mice showed that there was no improvement in the A740003-treated group compared with the control group (p > 0.05). The histological inflammatory scores of mice that received A740003 treatment were markedly higher than those of the sham group (p < 0.001). As shown in Fig. 2F, mice that received A740003 had significantly different macroscopic damage scores compared with those of the sham mice and control mice (p < 0.001). Next, intestinal samples from each animal were homogenized, and protein was extracted so that the myeloperoxidase (MPO) activity could be analyzed (Fig. 2G). The MPO activity was remarkably increased following BzATP administration and decreased when A740003 was administered compared to the control and sham animals (p < 0.001).

#### Intestinal permeability

**Figure 3:**
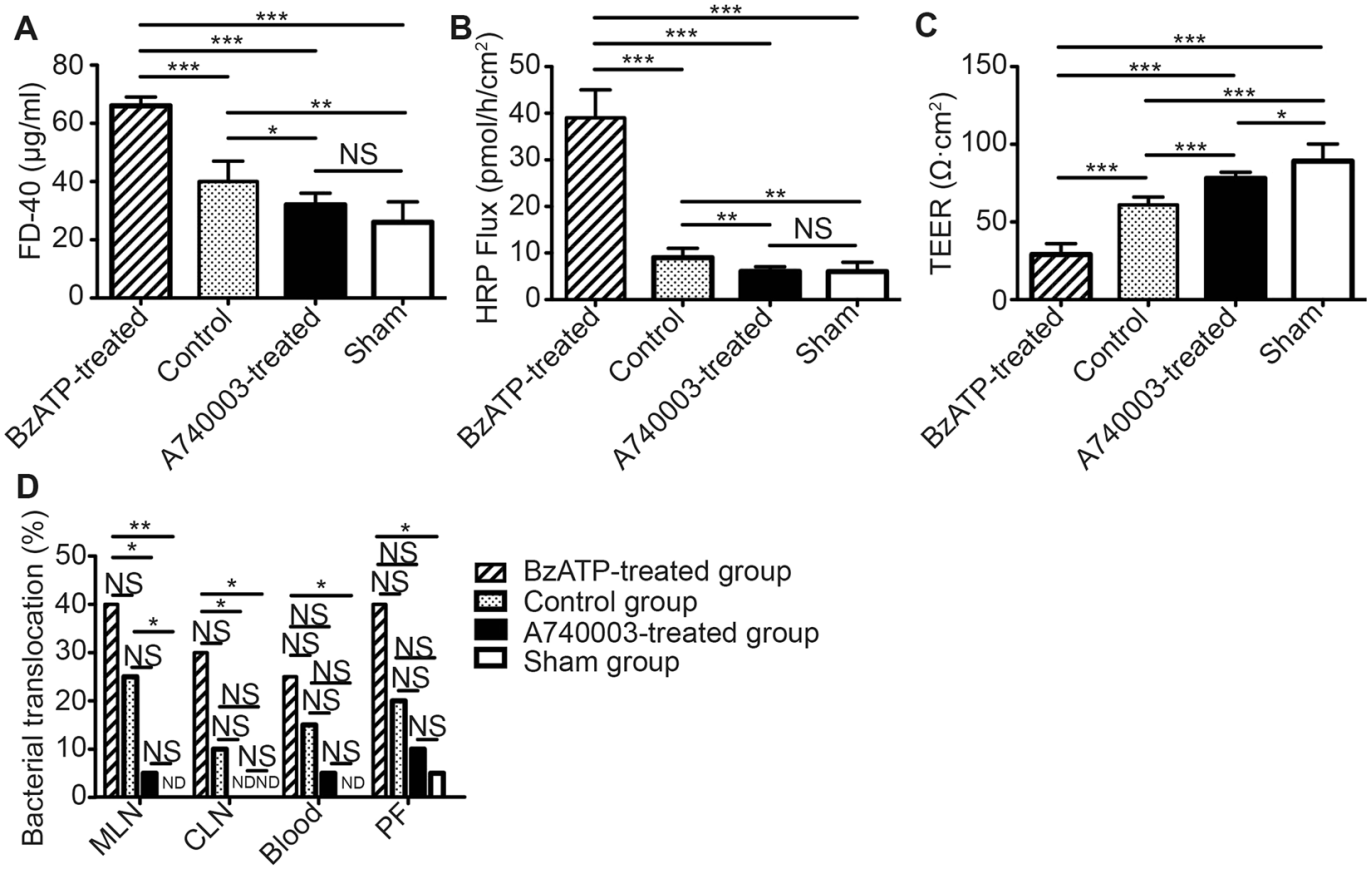
The effects of a P2X7R blockade on intestinal permeability and bacterial translocation. *In vivo* and *in vitro* permeability and bacterial translocation were assessed at 48 hours after CLP. (**A**) Mice in each group were fasted for 4 hours and then administered FD-40. Serum FD-40 was measured to evaluate *in vivo* permeability. (**B**,**C**) The *in vitro* permeability of the mouse intestines was measured by performing Ussing Chamber Analyses. (**D**) The MLN, CLN, blood, and PF were collected and cultured at 37 °C for 24 hours. The culture results of bacterial growth were considered positive when more than 10^2^ colonies/g of tissue were found. N = 6 mice per group. The data are expressed as the means ± SD. *p < 0.05, **p < 0.01 and ***p < 0.001 when compared among the different groups.

Intestinal permeability was measured both *in vivo* and *in vitro*. As shown in Fig. 3A and B, mice in the BzATP-treated group had higher levels of serum FD-40 and permeability compared with the sham group (p < 0.001) and control group (p < 0.001). The levels of FD-40 and intestinal permeability were significantly reduced in the A740003-treated group compared with those in the control group (p < 0.05, and p < 0.01, respectively); no differences were found between the A740003-treated mice and sham mice (p > 0.05). Additionally, mice in the A740003-treated group had increased small bowel permeability resistance when compared with the control group (p < 0.001), and the resistance was decreased compared with the sham group (p < 0.05; Fig. 3C). These data indicated that inactivation of P2X7R could decrease intestinal hyperpermeability induced by sepsis.

#### Bacterial translocation

P2X7R activation has been found to increase intestinal permeability, and this change may lead to bacterial translocation, which is known to play an important role in sepsis etiology^17^. Bacterial translocation to mesenteric lymph nodes (MLN), caudal lymph nodes (CLN), blood, and peritoneal fluid (PF) could be used as a readout for intestinal barrier integrity (Fig. 3D). CLP mice that received BzATP treatment exhibited obvious bacterial translocation to the MLN, CLN, blood, or PF compared with the sham group (p < 0.01, p < 0.05, p < 0.05, and p < 0.05, respectively), and there were no significant differences when compared with the control group (p > 0.05). No significant differences in bacterial translocation were found between the A740003-treated group and the sham or control groups (p > 0.05).

#### Apoptosis

Intestinal epithelial cell (IEC) apoptosis is thought to be one of the major accelerants of intestinal epithelial integrity disruption. As shown in Supplementary Fig. S2, the positive nuclei, indicated by red spots, were scattered diffusely in the field. The nuclei are fragmentary or pyknotic-like, and chromatin was aggregated in the areas surrounding the nuclei. IEC apoptosis in the BzATP-treated group was significantly increased compared with that of the sham group (p < 0.001) and control group (p < 0.001). In addition, apoptosis following A740003 treatment was significantly reduced compared with that in the control group (p < 0.001). There were no significant differences in apoptosis rates between the A740003-treated group and the sham group (p > 0.05).

#### The intestinal tight junction morphology

**Figure 4:**
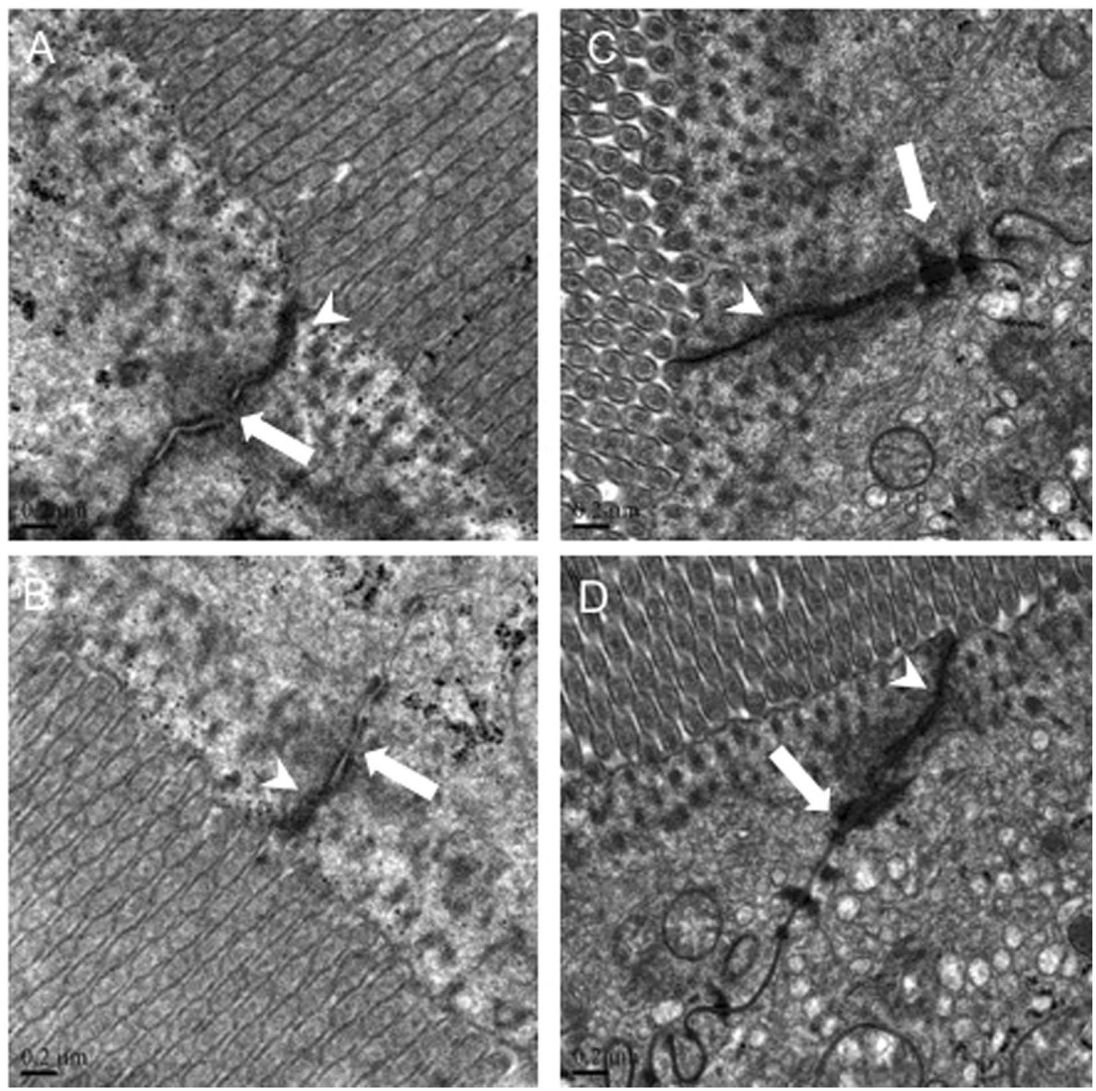
The effects of a P2X7R blockade on TJ morphology. Representative images of TJ morphology in the BzATP-treated (**A**), control (**B**), A740003-treated (**C**), and sham (**D**) groups assessed at 48 hours after CLP. Arrowheads indicate tight junctions and white arrows indicate desmosomes.

The effects of the P2X7R agonist and antagonist on intestinal histology have been verified by microscopy. We further examined the ultrastructural changes in the intercellular tight junction (TJ) by transmission electron microscopy (Fig. 4). The TJ stand and desmosome of the sham mice were clear and complete, and the paracellular space was narrow. In the control group of CLP mice, the microvilli were sparse and were irregularly long and arranged. With BzATP treatment, the TJ stand and desmosome were obscured or had disappeared, and the paracellular spaces were wider. In contrast, the amount of electron-dense materials in the TJ was increased and the desmosomes were clear in mice receiving A740003 treatment.

#### The intestinal tight junction proteins

**Figure 5:**
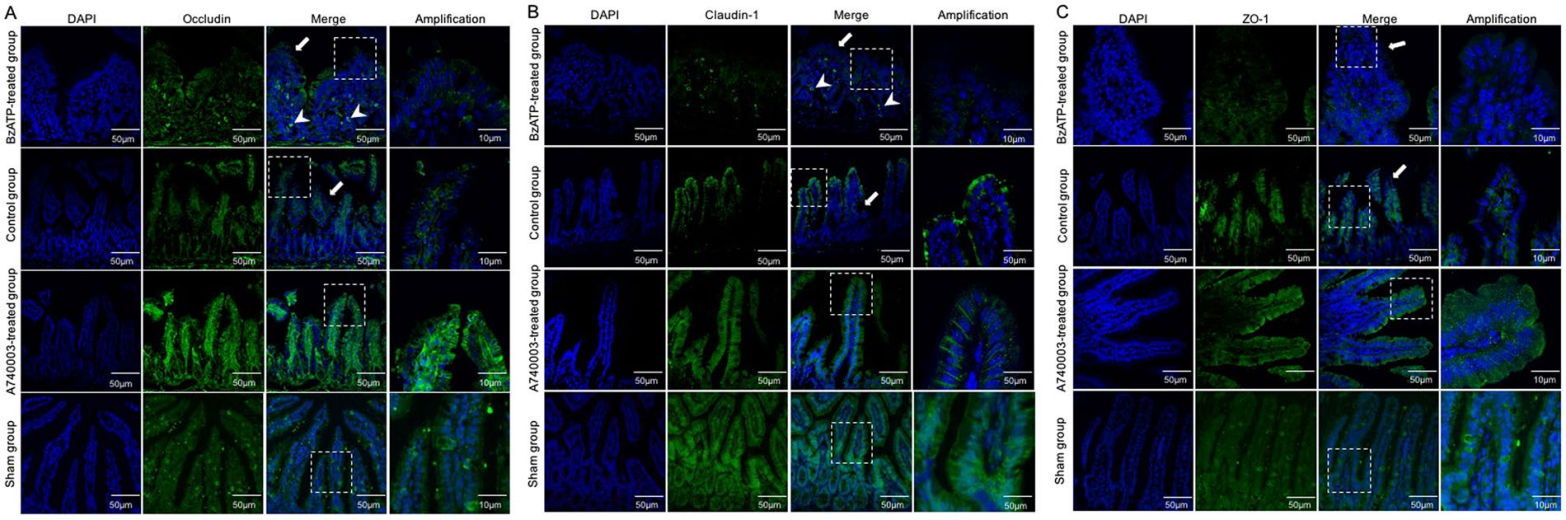
The effects of a P2X7R blockade on TJ distribution. Localization of occludin (**A**), claudin-1 (**B**), ZO-1 (**C**), and DAPI (DNA) within intestinal tissue sections assessed by immunofluorescence at 48 hours after CLP. TJ proteins (green) and DAPI stain (blue), merged TJ proteins and DAPI, as well as amplified merged TJ proteins and DAPI images are presented. Arrows indicate that biotin staining was ectopic to the lamina propria or deep within the epithelial surface. Arrowheads indicate the lack of focused staining on the surface of the villi.

**Figure 6:**
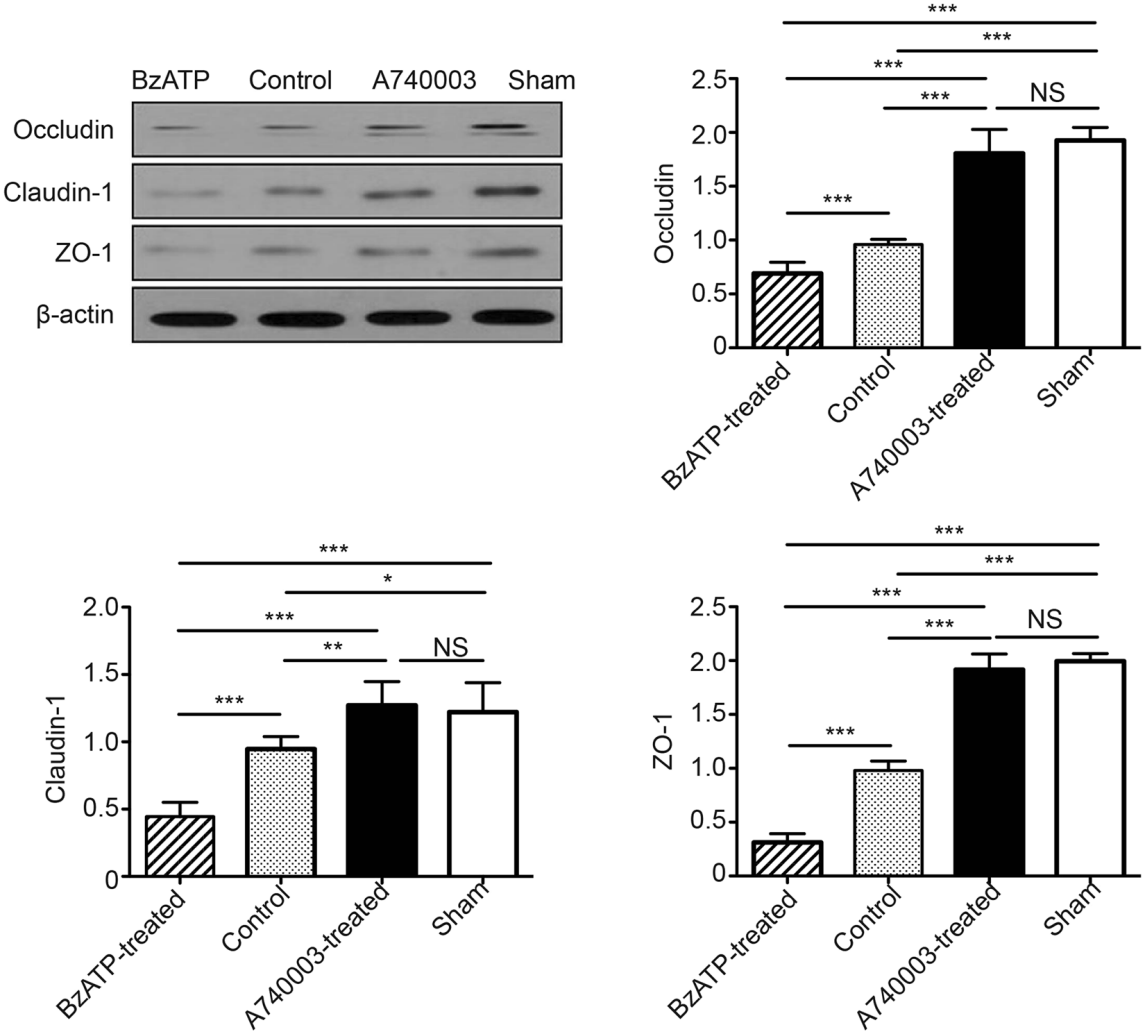
The effects of a P2X7R blockade on TJ expression. The protein levels of occludin, claudin-1, and ZO-1 in intestinal mucosa were measured by WB at 48 hours after CLP. N = 6 mice per group. The results of WB analysis are combined data from three densitometric scans (mean ± SD). *p < 0.05, **p < 0.01 and ***p < 0.001 when compared among the different groups.

Since TJ proteins can regulate intestinal epithelial permeability and play an important role in the maintenance of barrier function^18^, redistribution of those proteins could lead to altered TJ structure. In this study, both immunofluorescence and WB were used to evaluate TJ proteins of the intestinal mucosa (Figs 5 and 6). Biotin staining of occludin, claudin-1, and ZO-1 revealed a lack of focused staining in the lamina propria or deep within the surfaces of epithelial cells and some villi of CLP mice and BzATP-treated CLP mice, whereas a normal location and appearance of TJs were observed in the A740003-treated group. Quantitative analysis indicated that the concentrations of occludin, claudin-1, and ZO-1 were significantly higher in the A740003-treated group when compared with those of the control group (p < 0.001, p < 0.01, and p < 0.001, respectively). No differences were found between the mice receiving A740003 treatment and the sham mice (p > 0.05).

### Systemic blockade of P2X7R with A740003 inhibits M1 macrophages activation

**Figure 7:**
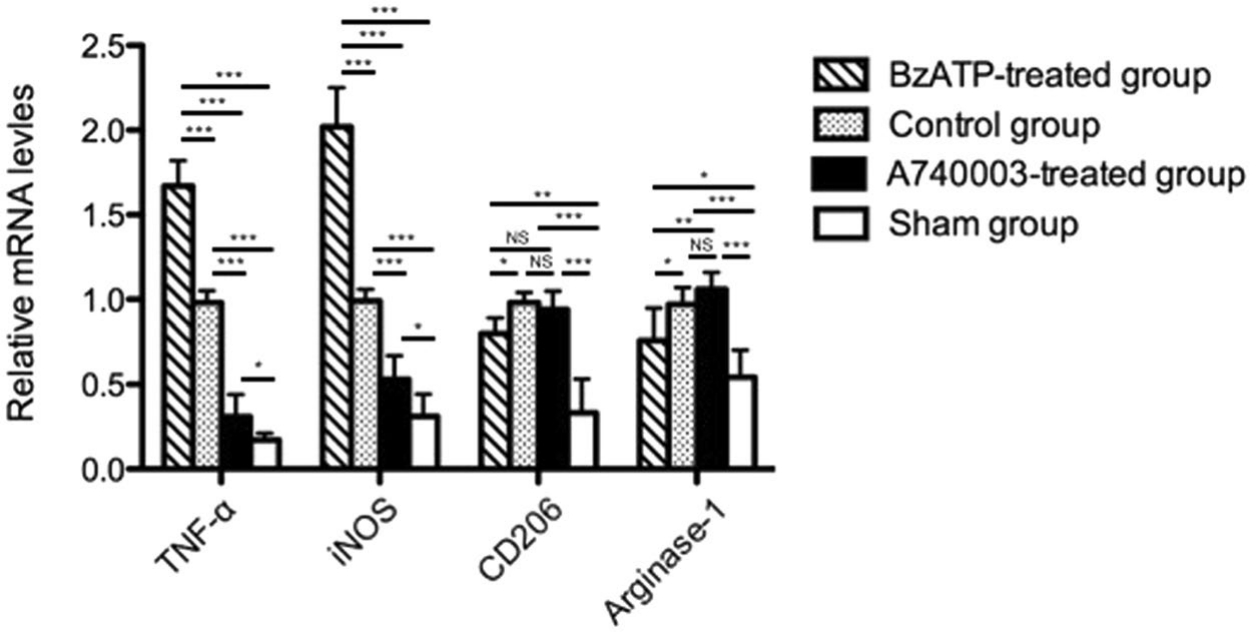
The effects of a P2X7R blockade on macrophage markers. The mRNA levels of M1-associated genes (TNF-α and iNOS) and M2-associated genes (CD206 and Arginase-1) in intestinal mucosa determined by quantitative PCR at 48 hours after CLP. N = 6 mice per group. The results are expressed as the means ± SD of three independent experiments. *p < 0.05, **p < 0.01 and ***p < 0.001 when compared among the different groups.

The above findings determined that a systemic blockade of P2X7R attenuated the production of pro-inflammatory cytokines (IL-6 and TNF-α) and protected against intestinal barrier disruption from sepsis-induced damage. However, the molecular mechanism behind these effects requires further research. Previous studies have shown that macrophages exhibit a spectrum of activation^7, 9^. Hence, we evaluated whether antagonists could affect macrophage activation. As shown in Fig. 7, A740003 injection caused macrophages to express significantly lower mRNA levels of M1-associated genes, including TNF-α and iNOS, when compared with those of the sham group (p < 0.05) and control group (p < 0.001). However, no remarkable differences in M2-associated genes, such as CD206 and Arginase-1, were found between the A740003-treated group and the control group (p > 0.05). The mRNA levels of CD206 and Arginase-1 were significantly higher in the A740003-treated mice than in the sham mice (p < 0.001). These data indicated that M1 macrophages activation was inhibited after administering a P2X7R blockade.

**Figure 8:**
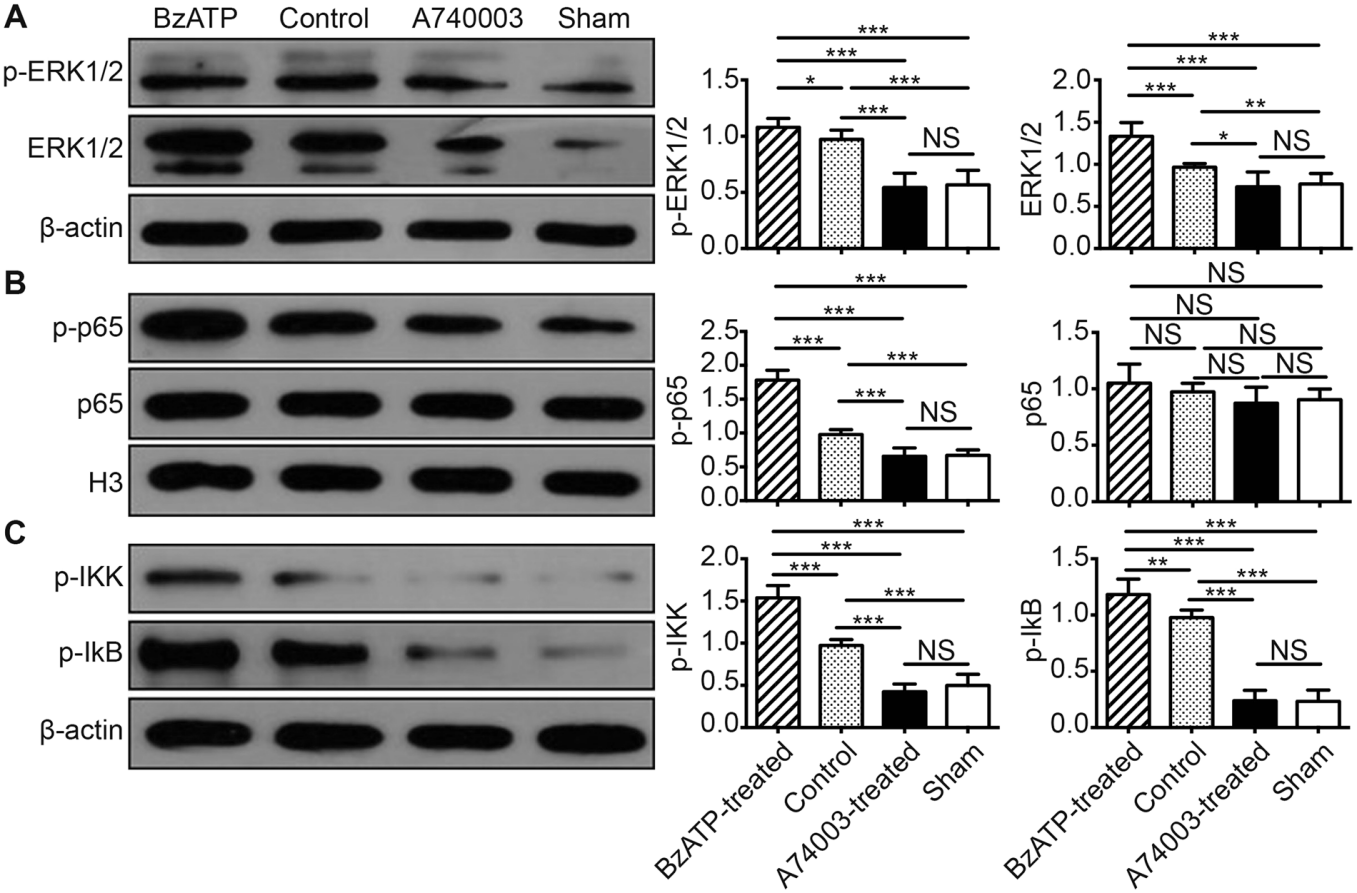
The effects of a P2X7R blockade on the ERK and NF-κB pathways. The protein levels of p-ERK 1/2 and ERK 1/2 (**A**), NF-κB p-p65 and p65 (**B**), and p-IKK and p-IκB (**C**) in intestinal mucosa were measured by WB at 48 hours after CLP. N = 6 mice per group. The results of WB analysis are combined data from three densitometric scans (means ± SD). *p < 0.05, **p < 0.01 and ***p < 0.001 when compared among the different groups.

ERK and NF-κB signals were detected in the following experiments. It was observed that p-ERK 1/2 and ERK 1/2 expression was significantly decreased in the A740003-treated group compared with the control animals (p < 0.001, and p < 0.05, respectively), and no differences were found between the A740003-treated group and the sham group (p > 0.05; Fig. 8A). The results presented in Fig. 8B and C show that blocking P2X7R reduced nuclear NF-κB immune content. The expression of p-p65, p-IKK, and p-IκB was remarkably decreased compared with the control group (p < 0.001), and there were no differences between the mice injected with A740003 and the sham mice (p > 0.05). No differences were found in the levels of the non-phosphorylated form of p65 among the groups (p > 0.05).

We further verified the effects of P2X7R antagonists *in vitro*. To examine whether agonists/antagonists interfere with P2X7R activity, we tested their effects on [Ca^2+^]_c_ and pore formation, two hallmarks of P2X7R activation, in primary macrophages (Supplementary Fig. S3). BzATP (500 μM) induced a strong, sustained increase in [Ca^2+^]_c_ in macrophages, as reported by many previous studies^19^. The increased [Ca^2+^]_c_ evoked by BzATP were significantly reduced in a concentration-dependent manner after treatment with various doses of Brilliant Blue G (BBG). Fitting the mean data with the Boltzmann equation yielded an IC_50_ of 0.74 μM. Another salient functional property of P2X7R is that prolonged activation of the receptor causes the formation of non-selective membrane pores, which enables the cells to uptake cationic fluorescent dyes, such as ethidium bromide (EB). Therefore, we subsequently examined the effects of BBG on membrane pore formation by detecting the BzATP-induced uptake of EB in primary macrophages. The EB uptake of macrophages induced by BzATP was also inhibited by BBG in a concentration-dependent manner, with an IC_50_ of 1.57 μM. BBG at 10 μM level significantly reduced the EB fluorescent intensity compared with the groups of other levels (p < 0.001) and the control group (p < 0.05). These data indicated that BBG (10 μM) had efficient inhibitory effects on BzATP-induced increased [Ca^2+^]_c_ and pore formation by antagonizing P2X7R in macrophages. Then we examined whether BzATP/BBG treatment could affect the activation of mouse lamina propria macrophages. The isolated cells were stimulated with 500 μM BzATP or 10 μM BBG for 30 min. As shown in Supplementary Fig. S4, BBG reduced the mRNA expression levels of M1-associated genes compared with the sham mice and control mice (p < 0.05 and p < 0.001). The expression of M2-associated genes was markedly higher in the BBG-treated group than in the sham group (p < 0.001). However, the levels of CD206 and Arginase-1 were not significantly different between the BBG-treated group and the control group (p > 0.05).

Taken together, based on the results of both *in vivo* and *in vitro* studies, the systemic P2X7R blockade could downregulate pro-inflammatory cytokine productions and maintain intestinal barrier function. The protective effects of P2X7R antagonists on intestinal barrier were partly through inhibiting M1 macrophages activation via ERK/NF-κB pathways.

## Discussion

In this study, we tested the therapeutic effects of P2X7R antagonists in polymicrobial sepsis, and we especially focused on their effects on the intestinal barrier. It is found that the systemic P2X7R blockade attenuated the production of pro-inflammatory cytokines and protected against intestinal barrier disruption. Specifically, we demonstrated that the protective effects of P2X7R antagonists were partly due to the inhibition of M1 macrophages activation via ERK/NF-κB signaling pathways, thereby mitigating the detrimental effects of M1 macrophages on the mucosal barrier integrity.

To our knowledge, there have been no studies focusing on the effect of P2X7R on sepsis-induced intestinal barrier damage. The intestinal barrier, composed of apical cell membranes from enterocytes and intercellular TJs, can prevent the invasion of harmful microorganisms, antigens and toxins from intestinal lumen into the blood. In this study, we demonstrated that blocking P2X7R could alleviate the damage of intestinal epithelial barrier in CLP septic animals. *In vivo* and *in vitro* measurements of intestinal permeability showed that the antagonists alleviated intestinal hyperpermeability in septic mice. The increased intestinal epithelial apoptosis was reduced after administering a P2X7R blockade. The expression levels of TJ proteins, such as claudin, occludin, and ZO-1, were also restored in mice that received the P2X7R antagonists. It has been reported that inflammatory cytokines can downregulate the intestinal barrier integrity^20, 21^. Hence, maintenance of epithelial integrity in the P2X7R antagonists-treated mice can be attributed to inhibition of pro-inflammatory cytokine secretion in the current study.

Additionally, we found that systemic administration of P2X7R antagonists resulted in decreased expression of M1 cell surface markers. Thus, we concluded that blocking P2X7R limits inflammatory responses partly by inhibiting M1 macrophages activation. Macrophages influence the duration, quality and magnitude of the innate immune response and balance the anti-pathogen response associated with potential tissue injury^22^. M1 and M2 macrophages differentially express a variety of effector molecules, including TNF-α, iNOS, CD206, and Arginase-1, that have distinct impacts on inflammatory outcomes^7^. In this study, we obtained evidence that, in CLP-induced septic mice, P2X7R antagonists could inhibit M1 macrophages activation, and thereby regulate the secretion of inflammatory cytokines and mitigate the detrimental effects on epithelial cell integrity.

In the pathophysiology of sepsis and septic shock, NF-κB activation is a central event that leads to the activation of complex cytokine and inflammatory mediator networks^23, 24^. The inhibition of NF-κB activation obtained by blocking P2X7R in CLP mice reinforced the findings from previous studies that suggested a beneficial effect of NF-κB repression^25, 26^. Another important intracellular signaling pathway controlled by the inflammatory response is the mitogen activated protein kinase (MAPK) pathway, which was recently shown to be activated by P2X7R signaling^27^. Here, we also showed that a P2X7R blockade effectively inhibited ERK activation in the intestinal mucosa. These results corroborate the existence of cross-talk between the MAPKs and NF-κB^28, 29^, supporting a mechanistic cooperation between various signaling pathways for the control of inflammatory responses.

We also tested the therapeutic effects of the P2X7R blockade on the survival of septic mice. No significant differences were found in mice receiving antagonists compared with the control animals in our study. However, the study by Csoka *et al*.^30^ showed that the survival rates of P2X7 knockout mice were significantly lower than CLP mice, indicating that the ATP interaction with P2X7R is protective. This discrepancy may be attributed to the inhibited inflammatory responses and mitigated intestinal epithelial barrier injuries upon the administration of antagonists in septic mice.

P2X7R expression in intestinal tissues and primary macrophages after agonist/antagonist stimulation was detected in this study. Interestingly, we observed that the systemic blockade by A740003 or activation by BzATP modulated the expression of P2X7R in a significant manner after CLP induction. Our finding were in accordance with the study of Wu *et al*.^31^, which indicated that P2X7R protein levels increased after BzATP stimulation in hepatic stellate cells. Their study also showed that A438079, another P2X7R antagonist, downregulated the expression of the receptor. Other antagonists of the receptor, such as BBG, were found to reduce the levels of P2X7R mRNA and protein in myocardial ischemic rats^32^. No available literature reported the effects of A740003 on P2X7R levels. The possible mechanism contributing to these effects may be due to membrane internalization stimulated by P2X7R under infection conditions. Feng *et al*. suggested that P2X7R activation stimulated internalization of the receptor, which could induce receptor redistribution, degradation and replenishment in the membrane, leading to increased receptor expression^33^. The administration of antagonists could block the effects of BzATP on decreased membrane capacitance, which is a reflection of receptor internalization^34^. However, no further explanations were provided in the current studies. The complex mechanisms behind the regulation require further research.

With respect to the agonists/antagonists used in this study, BzATP, A740003 and BBG concentrations and routes were based on previous evidence of systemic activation or inhibition of P2X7R^19, 35^. However, the two antagonists used in this study belong to different classes that have distinct chemical features. BBG has been described as having a slow rate of association and not competitively blocking agonist activation of P2X7R^36, 37^. Alternatively, A740003 has an increased affinity for selectively binding and competitively blocking P2X7R and has less time-dependent changes in potency compared with other antagonists, thus provides a wider therapeutic window^38, 39^. In addition, A740003, the selective antagonist, has a similar affinity for blocking P2X7R-mediated calcium influx, pore formation and releasing inflammatory cytokines^38, 40^. Therefore, successful treatment with A740003 corroborates the results of BBG and reinforces the idea of P2X7R as a therapeutic target in regulating intestinal integrity.

Several limitations of this study should be acknowledged. First, we found that intestinal barrier dysfunction could be alleviated by a P2X7R blockade through inhibition of M1 macrophages activation via ERK/NF-κB pathways. But more studies need to be conducted to elucidate the mechanisms since innate immunity is so complex. Second, no receptor binding assays were performed in this study. Only the activation or inactivation of P2X7R in intestinal macrophages was evaluated by WB or quantitative PCR analysis. Third, whether co-receptors are required for proper activation or blocking on the surface of macrophages is unclear. Understanding the complex and sequential molecular mechanisms by which macrophages are recruited to the intestines in response to bacteria remains an important goal to treat gut-origin sepsis. Additionally, the administration of P2X7R inhibitors suggests caution given the possibility of increasing the risk of tumor growth^41, 42^. The present study did not investigate the possible effects of P2X7R on the proliferation of IECs. Additional studies are required to provide a better understanding of the impacts of P2X7R on inflammation during host-tumor interactions to determine the safety of P2X7R antagonists.

In conclusion, these findings show that a systemic blockade of P2X7R mitigates inflammatory responses and maintains intestinal barrier function partly by inhibiting M1 macrophages activation via ERK/NF-κB pathways. Therefore, P2X7R could be a potential therapeutic target for the treatment of intestinal barrier dysfunction during sepsis.

## Materials and Methods

### Animals

Male 2-month-old C57BL/6 mice (each weighing between 20 and 25 g) obtained from the Animal Center of Jinling Hospital (Nanjing, China) were maintained under specific pathogen-free conditions in a temperature controlled room (24 °C) on a 12-hour/12-hour light and dark cycle. The institutional animal ethical committee of Jinling Hospital approved the animal study. The methods were carried out according to the approved guidelines.

### CLP

Polymicrobial sepsis was induced by CLP as described previously^43^. At the time of surgery, the mice were anesthetized by an intraperitoneal injection of 0.1 ml/10 g chloral hydrate (4%, Sigma-Aldrich, St Louis, MO, USA). After the laparotomy, a 4-0 silk ligature was placed 1.5 cm from the cecal tip. The cecum was punctured twice with a 21-G needle and gently squeezed to express an approximate 1 mm column of fecal material. The abdominal incision was closed in two layers with 4-0 silk sutures. After surgery, 1 ml of pre-warmed normal saline was given intraperitoneally. Mice were placed in individual cages and set on a warming pad.

### Experimental design

BzATP [3′-O-(4-benzoylbenzoyl) adenosine 5′-triphosphate] (Tocris Bioscience, Bristol, UK) was the P2X7R agonist used in this study, and it was injected through the intraperitoneal route at 5 mg/kg^44^. The P2X7R blocker utilized in this work was A740003 [N-(1-{[(cyanoimino)(5-quinolinylamino) methyl] amino}-2,2-dimethylpropyl)-2-(3,4-dimethoxyphenyl) acetamide] (Tocris Bioscience, Bristol, UK)^38^. A740003 was dissolved in distilled water and 40% hydroxypropyl-β-cyclodextrin and administered at 30 mg/kg. The doses of the compounds were determined by the preliminary experiments (Supplementary Information).

Twenty-four hours after CLP induction, the mice were randomly divided into three groups to test the effects of P2X7R agonists or antagonists on inflammatory responses and sepsis-induced intestinal barrier dysfunction. The first group was intraperitoneally injected with BzATP (BzATP-treated group) and the second group was injected with A740003 (A740003-treated group). The third group was injected with normal saline at 0.5 ml/mouse (control group). The animals were then maintained under specific pathogen-free conditions for an additional 24 hours. In addition, the normal control group (sham group) underwent sham surgery where the cecums were exteriorized without ligation and puncture as described previously. Plasma samples and the distal small bowel, beginning at 1 cm proximal to the ileocecal junction, were collected for histological analysis, organ culture, and biochemical studies (described below) 48 hours after CLP or sham operations. In the survival studies, the mice in the four groups were monitored for 48 hours to determine mortality rates.

### Plasma cytokine levels

The plasma levels of TNF-α, IL-6, IL-10, and IL-13 were evaluated using a commercially available solid-phase enzyme-linked immunosorbent assay (ELISA) kit (R&D Systems, Minneapolis, MN, USA).

### Organ culture and cytokine measurements

Intestinal explants were cultured in RPMI 1640 medium supplemented with 10% fetal bovine serum (FBS; Gibco-Invitrogen, Carlsbad, CA), 2 mM L-glutamine, 50 μM 2-mercaptoethanol, 10 mM HEPES, 100 U/ml penicillin and 100 μg/ml streptomycin (all from Sigma-Aldrich, St Louis, MO, USA) for 24 hours at 37 °C in a 5% CO_2_ humidified incubator. The samples were centrifuged and the supernatants were used to measure the concentrations of the cytokines TNF-α, IL-6, IL-10, and IL-13 using ELISA kits (Invitrogen, Camarillo, CA, USA).

### Macroscopic and histological assessment of intestinal injury

The intestinal tissues were obtained 48 hours after sepsis induction to evaluate the severity of inflammation both macroscopically and histologically in accordance with the criteria previously reported by Antonioli *et al*.^45^. The macroscopic criteria included the presence of adhesions between the intestines and other intra-abdominal organs, the consistency of the fecal material, thickening of the intestinal wall, and the presence and extension of hyperemia and macroscopic mucosal damage (assessed with the aid of a ruler). The microscopic evaluations were carried out using light microscopy. The ileum tissues were fixed in a 4% formaldehyde solution and embedded in paraffin wax using standard techniques. Slices (5 μm) were cut and stained with hematoxylin & eosin (H&E). For the histological assessment of intestinal injuries, a 0–4 grading scale was used as previously reported^46^. Two observers who were unaware of the group assignments performed all the damage assessments. The final score was expressed as a mean rank.

### MPO activity assessment

Intestinal samples were frozen at −80 °C until MPO was extracted. They were then homogenized in potassium phosphate buffer (pH 6.0), frozen and defrosted twice, homogenized again in potassium phosphate buffer (pH 6.0) containing 0.5% hexadecyltrimethyl-ammonium bromide (Sigma Chemical Co., St. Louis, MO, USA), and centrifuged at 40 000 *g* for 30 min at 4 °C. The supernatants were discarded and the insoluble pellets were re-homogenized in potassium phosphate buffer (pH 6.0) containing 0.5% hexadecyltrimethyl-ammonium bromide. Ten microliters of the supernatants was added to a 96-well plate containing 290 μl of 50 mM potassic PBS (pH 6.0), 3 μl of substrate solution containing 20 mg/ml o-dianisidine (Sigma Chemical Co., St. Louis, MO, USA), and 3 μl of H_2_O_2_ (20 mM). The plate components were rapidly mixed and the absorbance was determined at 460 nm for 1 min with a spectrophotometer. The MPO activity was measured using a standard curve constructed from the samples with units of MPO/g of intestinal sample. The total protein content of the biopsy specimens was estimated using the Pierce® BCA protein assay kit (Thermo Scientific, Rockford, IL, USA) and used to normalize the results.

### Measurement of intestinal permeability *in vivo* and *in vitro*

The mice were fasted for 4 hours and then administered fluorescein isothiocyanate (FITC)-dextran (FD-40; average molecular weight, 40 kDa; Sigma-Aldrich, St. Louis, MO, USA) by gavage at a dose of 600 mg·kg^−1^. After 4 hours, the mice were killed by cervical dislocation and bled by cardiac puncture. Serum was isolated using centrifugation, and serum FD-40 levels were evaluated using fluorometry. The *in vitro* permeability of the mice intestines was measured by performing Ussing Chamber Analyses as described previously^47^.

### Bacterial translocation

Using aseptic techniques, MLN (two samples for each mouse) and CLN were taken. After the collected tissue samples were weighed, 0.1 g of each node was homogenized in a tissue grinder with 0.9 ml of sterile saline. The homogenates were diluted, and 100 μl dilutions were cultured on Mac-Conkey’s agar (Sigma-Aldrich, St Louis, MO, USA) at 37 °C for 24 hours. Additionally, blood (obtained by cardiac puncture) and PF were obtained for bacterial colony counts by diluting and plating on LB agar (Sigma-Aldrich, St Louis, MO, USA) at 37 °C for 24 hours. Bacterial growth on the plates was expressed as colony-forming units/g of tissue. The culture results were considered positive when more than 10^2^ colonies/g of tissue were found^47^.

### Transmission electron microscopy of tight junctions

Slices of the distal ileum tissues (2 mm) were fixed for 2 hours in 4% buffered glutaraldehyde. The sections were cut into smaller pieces, post-fixed in 1% osmium tetroxide, sequentially dehydrated using graded alcohols, infiltrated using Epon 812 and then embedded in resin. Thin sections were cut and stained with uranyl acetate and lead citrate and examined with a Hitachi H-600 transmission electron microscope (Hitachi, Tokyo, Japan) operated at 75 kV.

### Immunofluorescence assessment of TJ proteins

The localization of occludin, claudin-1, ZO-1 proteins, and NF-κB-p65 was determined by immunofluorescence. The intestinal tissues were immediately removed, washed with PBS, mounted in embedding medium, and stored at −80 °C until use. Frozen sections (10 μm) were cut and mounted on slides. The 1:100 dilutions of rabbit polyclonal antibodies against occludin, claudin-1, ZO-1 (all from Abcam, Cambridge, UK), and NF-κB-p65 (Santa Cruz Biotechnology, Dallas, TX, USA) were incubated according to the manufacturer’s instructions. The sections were probed with their respective FITC-conjugated secondary IgG antibodies. The nuclei were counterstained with DAPI (4, 6-diamidino-2-phenylindole). Slides incubated in the absence of primary antibodies were used as negative controls. Confocal analysis was performed with a confocal scanning microscope (Leica Microsystems, Heidelberg GmbH, Mannheim, Germany).

### Terminal deoxynucleotidyl transferase dUTP nick end labeling (TUNEL) staining

Apoptotic cells in the intestinal sections were identified according to the TUNEL staining method using a commercial kit (Roche Diagnostics Corp., Indianapolis, IN, USA) according to the manufacturer’s protocol. The apoptotic index was defined as the percentage of TUNEL-positive cells (red stain) in 100 randomly chosen intraepithelial cells as previously described^48^.

### Isolation of lamina propria macrophages

Lamina propria macrophages from the intestinal mucosa of septic mice were isolated as previously reported^49, 50^. Briefly, the tissues were first predigested to eliminate epithelial cells. The intestinal tissues were then digested with digestion solution (1 mg/mL collagenase type VIII + 3 mg/mL dispase II + 40 μg/mL DNase I + 5% FBS) + Hanks Solution (all reagents were from Sigma-Aldrich, St Louis, MO, USA). After incubation at 37 °C for 30 min while shaking (150 rpm), the tissues were filtered through a 100-μm nylon mesh filter (Fisher brand, Loughborough, UK). The remaining pieces were repeatedly digested and filtered, and the filtered suspensions were rinsed with PBS. The cell suspensions were centrifuged, resuspended in fresh PBS, and filtered again through a 40-μm nylon mesh filter (Fisher brand, Loughborough, UK).

### Cell culture and intervention

Macrophages isolated from intestinal mucosa of mice 24 hours after CLP induction were grown in RPMI-1640 medium containing 10% FBS, 2 mM L-glutamine, 100 U/ml penicillin and 100 μg/ml streptomycin. The cells were grown at 37 °C in a humidified atmosphere with 5% CO_2_. Measurement of [Ca^2+^]_c_ and EB dye uptake in macrophages were conducted as previously described^19, 51^. The antagonist group received BBG (Sigma-Aldrich, St Louis, MO, USA), a potent P2X7R antagonist^52^, at a final concentration of 10 μM, and the agonist group received BzATP to activate P2X7R expression (500 μM final concentration). The control group did not receive any supplements. The cultured cells in each group were prepared for further analysis 30 min after incubation.

### Quantitative PCR analysis

The mRNA levels of P2X7R, TNF-α, iNOS, CD206, and Arginase-1 expressed in the intestinal mucosa or the isolated macrophages were measured by quantitative PCR analysis. Briefly, after total RNA was extracted from intestinal mucosa tissues using TRIZOL reagent (Life Technologies Inc., Carlsbad, CA, USA), the oligo (dT)-primed complementary DNA was used for reverse transcription of the purified RNA. The transcript amounts of the genes of interest were measured by quantitative PCR using SYBR Green detection (Applied Biosystems, Carlsbad, CA, USA). Expression levels of each gene were expressed as a relative value to GAPDH and then normalized to the mean value of the control group^47^. All reactions were independently repeated three times to ensure reproducibility. The primer sequences are listed in Supplementary Table S1.

### WB analysis

The intestinal tissues were cut open and washed with PBS after the mice were sacrificed, and then the intestinal mucosa was harvested and homogenized in radioimmunoprecipitation assay buffer (Beyotime Institute of Biotechnology, Haimen, China). The supernatant was used to the determine protein concentration via a bicinchoninic acid assay (Beyotime Institute of Biotechnology, Haimen, China). Equal amounts of extracts were separated by 10% SDS-PAGE, transferred onto polyvinylidene difluoride membranes (Millipore, Billerica, MA, USA), and then blotted. GAPDH, H3, or β-actin was used as an internal control. The antibodies used in the present study included 1:1000 dilutions of rabbit polyclonal antibody against P2X7R, p-ERK, p-ERK1/2, p65, p-p65, p-IKK, p-IκB, occludin, claudin-1, or ZO-1 (Abcam, Cambridge, UK). Quantification was performed by optical density methods using ImageJ software. The results were expressed as relative density to GAPDH, β-actin, or H3 and then normalized to the mean value of the control group^47, 53^.

### Statistical analysis

The results are shown as means ± standard deviation (SD). Comparisons of continuous variables between groups were conducted using the Student’s t‑test or one‑way analysis of variance. The log-rank tests were used to compare differences in survival rates. Graphpad Prism software 6.0 (Graphpad Software Inc., La Jolla, CA, USA) and SPSS 17.0 (SPSS, Inc., Chicago, IL, USA) were used to perform the data analysis. p < 0.05 indicated a statistically significant difference.

## Electronic supplementary material

supplementary information
