# Supplementary material for: Systemic blockade of P2X7 receptor protects against sepsis-induced intestinal barrier disruption
Source: Sci Rep. 2017 Jun 29;7:4364. doi: 10.1038/s41598-017-04231-5 (PMC5491531; doi:10.1038/s41598-017-04231-5)
Supplement: Supplementary file 1 — supplementary information [file 41598_2017_4231_MOESM1_ESM.pdf]

Supplementary Information

Systemic blockade of P2X7 receptor protects against sepsis-induced intestinal barrier disruption

Xiuwen Wu, MD, Jianan Ren, MD, FACS*, Guopu Chen, MD, Lei Wu, MD, Xian Song, MS, Guanwei Li, MD, Youming Deng, MD, Gefei Wang, MD, Guosheng Gu, MD, Jieshou Li, MD

Department of Surgery, Jinling Hospital, Medical School of Nanjing University, Nanjing, China

Tables

Table S1. Primers used for quantitative PCR analysis

Genes	Primers sequence
P2X7R	F: 5'-AGCACGAATTATGGCACCGT-3’
	R: 5'-CCCCACCCTCTGTGACATTCT-3'
TNF-α	F: 5’-GAGTGACAAGCCTGTAGCC-3’
	R: 5’-CTCCTGGTATGAGATAGCAAA-3’
iNOS	F: 5’-CACCAAGCTGAACTTGAGCG-3’
	R: 5’-CGTGGCTTTGGGCTCCTC-3’
CD206	F: 5’-TTGGACGGATAGATGGAGGG-3’
	R: 5’-CCAGGCAGTTGAGGAGGTTC-3’
Arginase-1	F: 5’-CCAGAAGAATGGAAGAGTCAGTGT-3’
	R: 5’-GCAGATATGCAGGGAGTCACC-3’
GAPDH	F: 5’-CTTTGTCAAGCTCATTTCCTGG-3’
	R: 5’-TCTTGCTCAGTGTCCTTGC-3’

Figures and Figure Legends

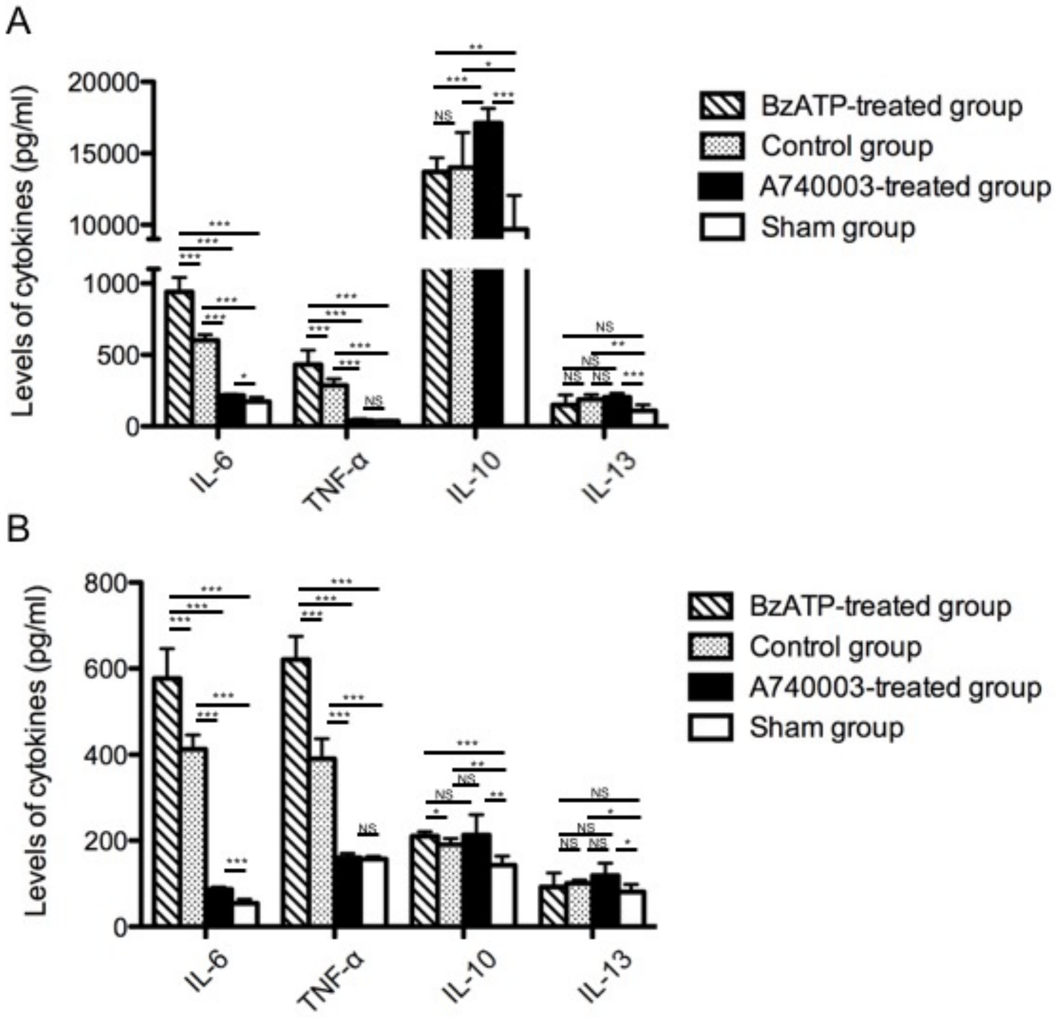

Figure S1. The effects of a P2X7R blockade on cytokine production. (A) Serum levels of the inflammatory cytokines IL-6, TNF-α, IL-10, and IL-13 were determined by ELISA at 48 hours after CLP. (B) The intestinal mucosal levels of the inflammatory cytokines IL-6, TNF-α, IL-10, and IL-13 were determined by ELISA at 48 hours after CLP. N=6 mice per group. The results are expressed as the means ± SD of three independent experiments. *$p < 0.05$, **$p < 0.01$ and ***$p < 0.001$ when compared among the different groups.

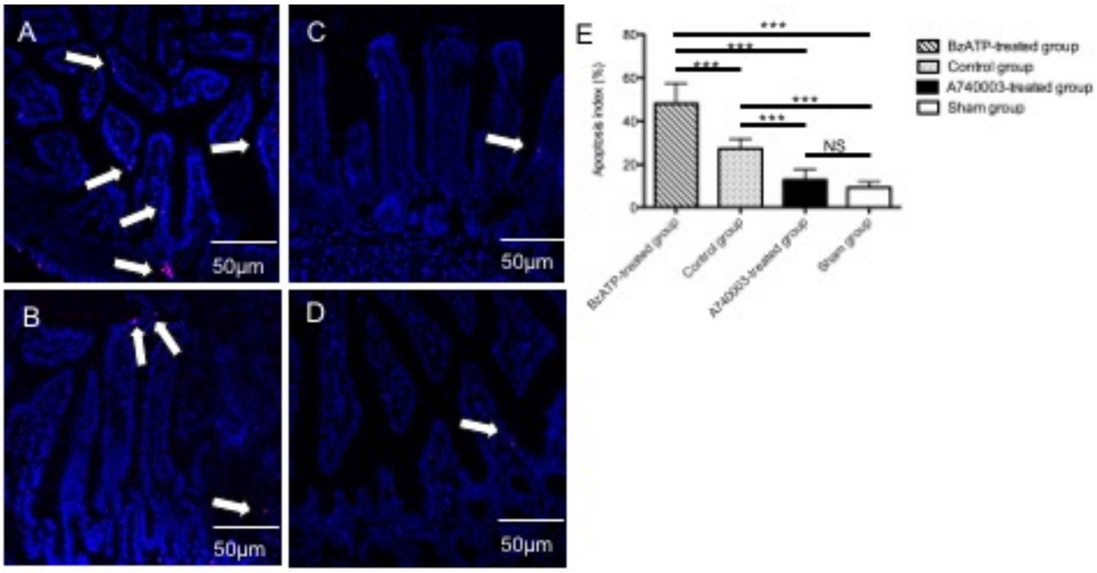

Figure S2. The effects of a P2X7R blockade on intestinal epithelial cell apoptosis. Representative TUNEL-stained intestinal sections in the BzATP-treated (A), control (B), A740003-treated (C), and sham (D) groups at 48 hours after CLP. The apoptosis index was demonstrated in (E) (mean ± SD). N=6 mice per group. *p < 0.05, **p < 0.01 and ***p < 0.001 when compared among the different groups.

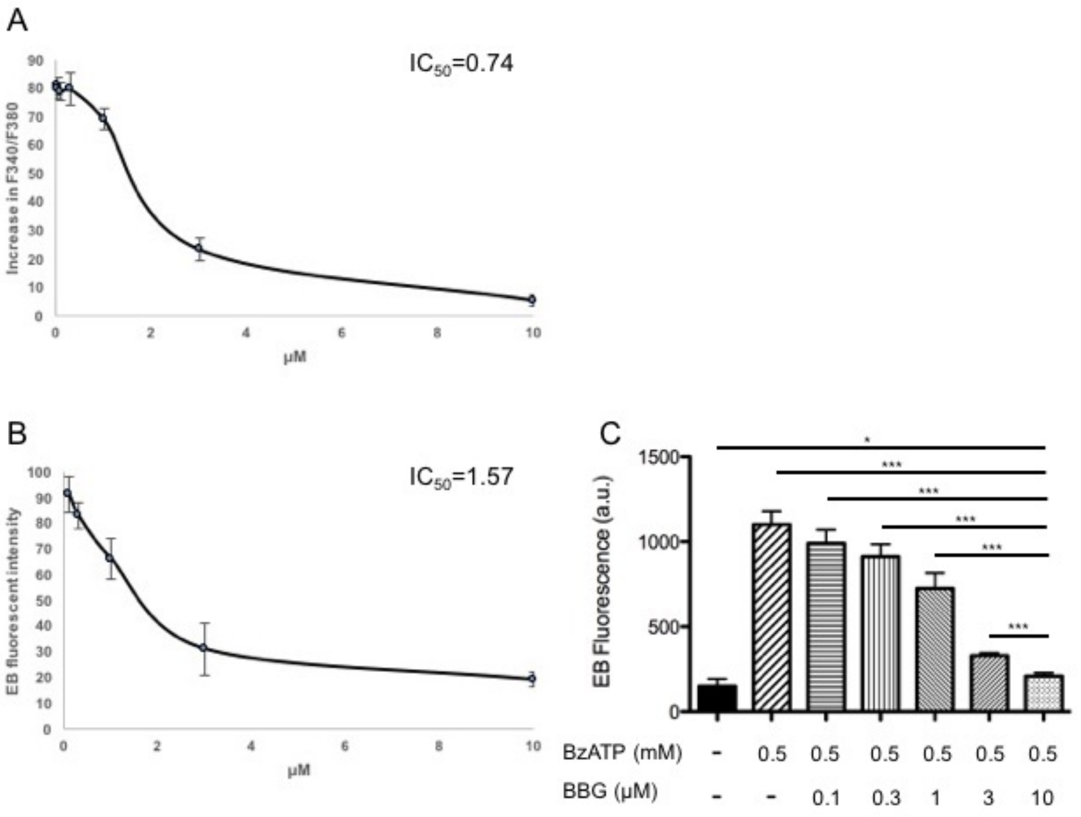

Figure S3. The effects of a P2X7R blockade on BzATP-induced increased $[Ca^{2+}]_c$ and pore formation in primary macrophages. (A) The statistical peak values of an increase in the F340/F380 ratio after the administration of BzATP alone and in the presence of BBG (0.01, 0.03, 0.1, 0.3, 1, 3, 10 μM) (n=20 cells in each case). The smooth curve represents the fit with the Boltzmann equation. (B) The statistical values of the relative EB uptake rates (BzATP by itself was taken as 100%). The smooth curve represents the fit with the Boltzmann equation. (C) Summary of the EB fluorescence intensity measured in each experimental group (n=150 cells in each case). The data are expressed as the means ± SD. *p < 0.05, **p < 0.01 and ***p < 0.001 when compared among the different groups.

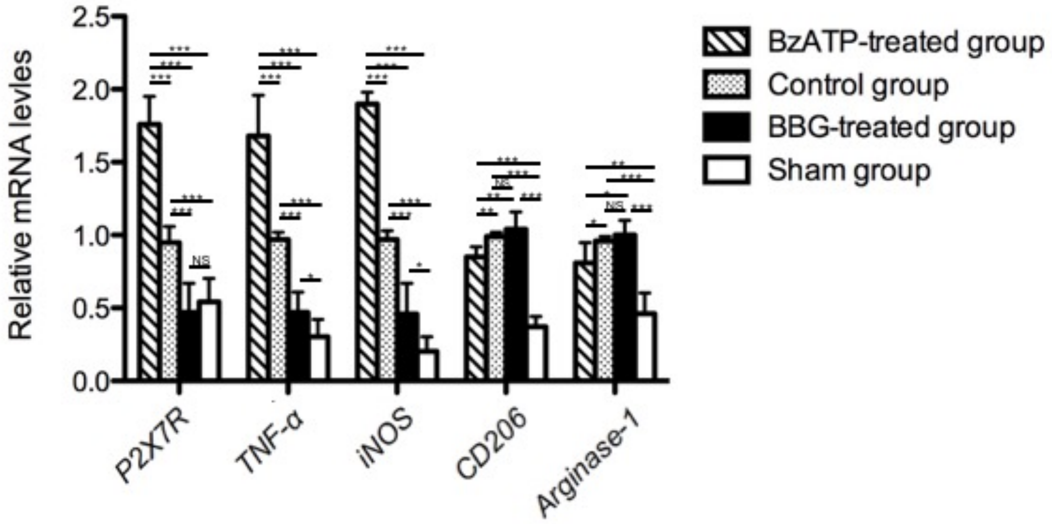

Figure S4. The effects of a P2X7R blockade on receptor expression and macrophage markers. The mRNA levels of P2X7R, M1-associated genes (TNF-α and iNOS), and M2-associated genes (CD206 and Arginase-1) in isolated primary macrophages were determined by quantitative PCR 30 minutes after BzATP/BBG stimulation. The results are expressed as the means ± SD of three independent experiments. $^*p < 0.05$, $^{**}p < 0.01$ and $^{***}p < 0.001$ when compared among the different groups.

Preliminary experiments

Male 2-month-old C57BL/6 mice underwent CLP to mimic polymicrobial sepsis. After 24 hours of CLP, the mice were randomly divided into six groups according to the received drug doses (N=6 in each group): 2.5, 5, or 10 mg/kg BzATP and 15, 30, or 60 mg/kg A740003. The control group was injected with normal saline (0.5 ml/mouse) (N=6). Intestinal tissues were obtained at 48 hours after CLP or sham surgery. A histological assessment was conducted to evaluate whether the severity of inflammation was aggravated or alleviated in each group. The BzATP dose was the minimal dose that induced the highest intestinal injury scores, whereas the A740003 dose was the lowest dose that mitigated intestinal injuries.

We found that the mice that received 5 mg/kg BzATP had significantly higher histological scores than those that received 2.5 mg/kg BzATP ($p < 0.05$) and the control group ($p < 0.01$). No significant differences were found between the groups that received 5 or 10 mg/kg ($p > 0.05$). A dose of 5 mg/kg was chosen for the systemic administration of BzATP. The administered dose of A740003 was determined to be 30 mg/kg. Mice injected with 30 mg/kg A740003 had significantly reduced intestinal injury scores compared with those injected with 15 mg/kg ($p < 0.05$) and the control group ($p < 0.01$). No significant differences were found between the groups that received 30 or 60 mg/kg A740003 ($p > 0.05$).

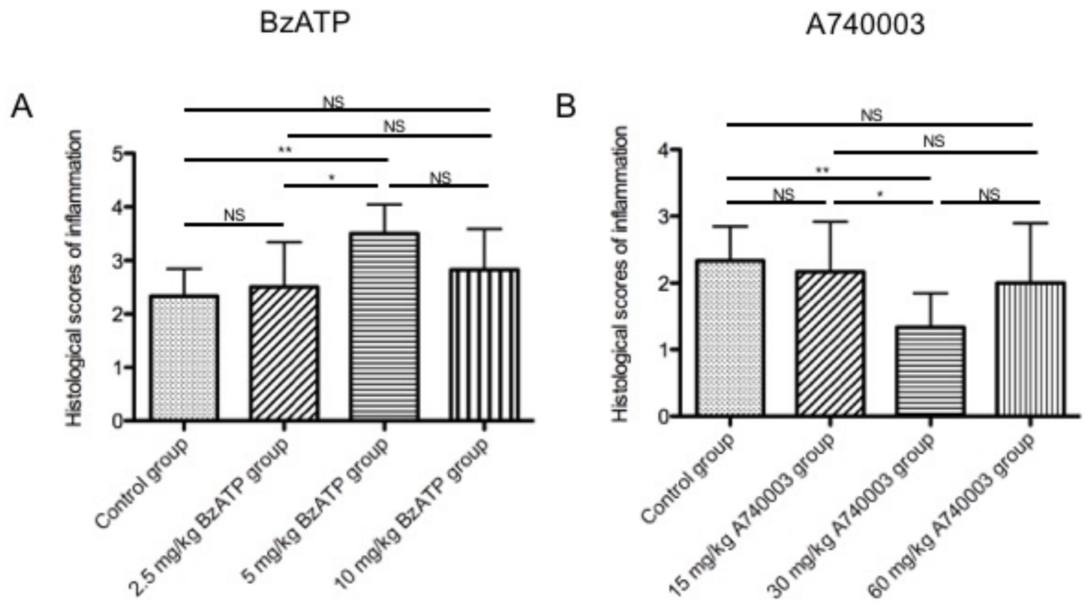

Figure S5. Histological scores of mice that received various doses of agonist or antagonist. (A) BzATP, at 2.5, 5, or 10 mg/kg, was administered through intraperitoneal injection to mice in each group at 24 hours after CLP. The control group was injected with normal saline (0.5 ml/mouse). The intestinal tissues were obtained 48 hours after sepsis induction to evaluate the severity of inflammation. The histological scores are expressed as the means ± SD. N=6 mice per group. *$p < 0.05$, **$p < 0.01$ and ***$p < 0.001$ when compared among the different groups. (B) A740003, at 15, 30, or 60 mg/kg, was injected intraperitoneally to mice in each group at 24 hours after CLP. The control group was injected with normal saline (0.5 ml/mouse). The intestinal tissues were obtained 48 hours after sepsis induction to evaluate the severity of inflammation. The histological scores are expressed as the means ± SD. N=6 mice per group. *$p < 0.05$, **$p < 0.01$ and ***$p < 0.001$ when compared among the different groups.
